# Identification of Differentially Expressed Proteins in Porcine Alveolar Macrophages Infected with Virulent/Attenuated Strains of Porcine Reproductive and Respiratory Syndrome Virus

**DOI:** 10.1371/journal.pone.0085767

**Published:** 2014-01-21

**Authors:** Yan-Jun Zhou, Jian-Ping Zhu, Tao Zhou, Qun Cheng, Ling-Xue Yu, Ya-Xin Wang, Shen Yang, Yi-Feng Jiang, Wu Tong, Fei Gao, Hai Yu, Guo-Xin Li, Guang-Zhi Tong

**Affiliations:** 1 Division of Swine Infectious Diseases, Shanghai Veterinary Research Institute, CAAS, Shanghai, China; 2 Department of Microbiology and Molecular Genetics, Michigan State University, East Lansing, Michigan, United States of America; Virginia Polytechnic Institute and State University, United States of America

## Abstract

The highly pathogenic porcine reproductive and respiratory syndrome virus (HP-PRRSV) is still a serious threat to the swine industry. However, the pathogenic mechanism of HP-PRRSV remains unclear. We infected host porcine alveolar macrophages (PAMs) with the virulent HuN4 strain and the attenuated HuN4-F112 strain and then utilized fluorescent two-dimensional difference gel electrophoresis (2D-DIGE) to screen for intracellular proteins that were differentially expressed in host cells infected with the two strains. There were 153 proteins with significant different expression (*P*<0.01) observed, 42 of which were subjected to mass spectrometry, and 24 proteins were identified. PAM cells infected with the virulent strain showed upregulated expression of pyruvate kinase M2 (PKM2), heat shock protein beta-1 (HSPB1), and proteasome subunit alpha type 6 (PSMA6), which were downregulated in cells infected with the attenuated strain. The upregulation of PKM2 provides sufficient energy for viral replication, and the upregulation of HSPB1 inhibits host cell apoptosis and therefore facilitates mass replication of the virulent strain, while the upregulation of PSMA6 facilitates the evasion of immune surveillance by the virus. Studying on those molecules mentioned above may be able to help us to understand some unrevealed details of HP-PRRSV infection, and then help us to decrease its threat to the swine industry in the future.

## Introduction

Porcine reproductive and respiratory syndrome (PRRS) is an infectious disease characterized by reproductive failure in female pigs and respiratory tract illness in young pigs [1], [2]. The highly pathogenic porcine reproductive and respiratory syndrome (HP-PRRS) that recently emerged in China is associated with additional symptoms, such as a high fever in infected pigs, high morbidity, and high mortality [3]–[5]. The pathogen causing the syndrome is the porcine reproductive and respiratory syndrome virus (PRRSV), which is an enveloped, non-segmented, single-stranded positive-sense RNA virus belonging to the family *Arteriviridae*, order Nidovirales [6], [7]. Based on antigenic and genetic differences, PRRSV isolates are divided into two distinct genotypes: the European type and the North American type [8]. The pandemic PRRSV strains in China primarily correspond to the North American type. PRRSV exhibits strict cell tropism, and porcine alveolar macrophages (PAMs) are the main target cells that become infected *in vivo*. PRRSV can replicate in infected PAMs [9]–[11] and cause changes in the morphology and function of PAM cells [12], [13].

In recent years, many new discoveries have been made concerning host cell changes at the molecular level that are caused by PRRSV infection. Technologies such as gene chips have been utilized to investigate changes in the host transcriptome following PRRSV infection, and it was found that PRRSV infection of host cells could cause changes in a large number of host genes at the transcriptional level [14]–[17]. Because there are certain differences between changes at the mRNA level and the actual protein expression level, several proteomic technologies have also been applied in the investigation of PRRSV infections. Zhang et al [18] utilized the JXwn06 strain of the virus (HP-PRRSV) to infect PAM cells *in vitro* and identified 23 differentially expressed proteins using two-dimensional electrophoresis. Ding et al. [19] utilized the JL/07/SW strain (HP-PRRSV) to infect PAM cells *in vitro* and detected 29 differentially expressed proteins. Xiao et al. [20] identified 45 differentially expressed proteins using the virulent Chinese strain and the attenuated North American strain to infect pigs *in vivo* and then compared the proteomic changes induced by the virulent/attenuated strain in lung tissue. However, the specific proteins involved in viral replication in these infections remain unknown. In addition, although the virulent HP-PRRSV strain that is pandemic in China and the classic North American attenuated strain both belong to the North American type of the virus, there are significant genomic sequence differences between the two strains. In particular, the highly pathogenic virulent strain carries deletions of 1 and 29 amino acids (aa) in the Nsp2 gene [4], [5], which are unrelated to the highly virulence [21]. The evolutionary history of the genetic backgrounds of the two strains remains unclear, which makes it difficult to explore the differences between PRRSV virulent and attenuated strains.

In contrast, the virulent HuN4 strain of HP-PRRSV isolated in our laboratory can cause disease and death in infected pigs [3], [5], and the continuous passage of this strain generated the attenuated vaccine strain HuN4-F112, which can provide protective immunity in pigs. This pair of virulent/attenuated strains has a relatively clear genetic background [22], [23], thus providing better experimental material for our investigation of the pathogenic differences between HP-PRRSV virulent/attenuated strains at the cellular level using two-dimensional difference gel electrophoresis (2D-DIGE) technology. This study employed this pair of virulent/attenuated strains to infect PAM cells to identify differentially expressed proteins. We annotated the Gene Ontology (GO) of the differentially expressed proteins, built a protein-protein interaction network through protein interaction network analysis, and, finally, validated the differentially expressed proteins *via* real-time PCR.

## Materials and Methods

### Ethics statement

The animal studies were approved by the Animal Care and Use Committee of Shanghai Veterinary Research Institute, CAAS.

### Virus and experimental pigs

Both the virulent HP-PRRSV strain HuN4 and the attenuated vaccine strain HuN4-F112 were isolated, passaged, and preserved in our laboratory [3], [5], [23]. Newborn piglets were purchased from the Experimental Animal Center, School of Agriculture and biology, Shanghai Jiao Tong University. The ethics committee of the college approved the study.

### Preparation of PAMs

Healthy newborn piglets were selected for isolation of PAMs based on a previously reported method [11]. PAM cells harvested from wash fluid were cultured in RPMI-1640 medium (Gibco Invitrogen, Shanghai, China) containing 10% fetal bovine serum (FBS) (Gibco), 100 units/mL penicillin, and 100 µg/mL streptomycin (Gibco) in cell incubators at 37°C with 5% CO_2_.

### Virus inoculation and sample preparation

When the PAM cell density exceeded 95% confluence, the cells were washed three times with phosphate buffered saline (PBS) and 2×10^6^ PAM cells were then inoculated with 10^3^ TCID_50_ (50% tissue culture infective dose) of either the HuN4 strain or the HuN4-F112 strain. PAM cells without virus were used as the control. After 1 h of infection, the culture medium was discarded and replaced with RPMI-1640 medium containing 2% FBS. The cell culture was continued, and periodic observations of the cytopathic effects were performed. The culture medium was discarded 48 h after inoculation. After gently washing the culture plate three times with pre-cooled sterilized PBS (pH 7.2–7.4) at 4°C, the PBS was discarded and the culture plate was placed on ice. Lysis buffer (pH 8.5) containing 7 M urea, 2 M thiourea, 65 mM Tris, 2% dithiotreitol (DTT), 4% 3-[(3-cholamidopropyl) dimethylammonio] propanesulfonate (CHAPS), 0.2% IPG buffer (GE Healthcare, Munich, Germany), and 0.1% v/v protease inhibitor mixture was added into each well. All the cells were scraped into a 1.5 mL centrifuge tube and homogenized with a Dounce's homogenizer. The cells were disrupted by sonication (80W, five times for a 10 s interval each time at 15 s intervals). All procedures were performed on ice. The mixture was centrifuged at 15,000 g for 45 min at 4°C. Protein content in the supernatants was determined by Bradford assays (Bio-Rad, USA). Samples of aliquots were stored at −80°C until use for proteomic analysis.

### Protein labeling

Three protein samples were labeled using the CyDye DIGE Fluor Minimal Labeling Kit (GE Healthcare, Munich, Germany). The dyes were designed to ensure that a given protein originating from different samples would have the same relative mobility regardless of the dye used to tag them. Fifty micrograms of an internal standard (IS) containing an equal amount of three protein samples collected at 48 h post-inoculation (hpi) were labeled with 400 pmol Cy2. And fifty micrograms protein sample of group A, B, and C were labeled with 400 pmol Cy3 or Cy5 using the CyDye DIGE Fluor Minimal Labeling Kit (GE Healthcare, Munich, Germany) according to the manufacturer's instructions. Details of the experimental design using the three-dye approach is illustrated in Table 1. Briefly, each CyDye minimal dye was redissolved in fresh N, N-dimethylformamide (DMF). To label proteins, 400 pmol of fluorescent dye was used to label 50 µg of protein at a pH of 8.5. The labeling reaction was carried out on ice for 40 min in the dark. Termination of the reaction was also carried out in the dark *via* reaction with 1 µL of 10 mM lysine for 10 min.

**Table 1:** Experimental design for protein labeling.

Gel No.	Cy2(blue)	Cy3(green)	Cy5(red)
1	IS	A	B
2	IS	B	A
3	IS	A	C
4	IS	B	C
5	IS	C	A
6	IS	C	B

Samples from the non-infected control group (A), samples infected by the HuN4 strain (B) or samples infected by the HuN4-F112 strain (C). IS, Internal Standard.

### 2D-DIGE

For each gel, Cy2-, Cy3-, and Cy5-labeled proteins (50 µg each) were combined and an equal volume of rehydration buffer (8 M urea, 4% w/v CHAPS, 130 mM DTT, and 2% v/v Pharmalyte™ pH 3–10) was added. The pooled protein samples were subjected to isoelectric focusing carried out on nonlinear IPG strips, length 13 cm, pH 3–10 (GE Healthcare), rehydrated at 30 V for 12 h at room temperature. Isoelectric focusing was conducted at 500 V for 1 h, followed by 1000 V for 1 h, then 8000 V for 3 h and held at 8000 V to reach a total of 40000 Vh at 20°C and a maximum current setting of 50 µA per strip using Ettan IPG-phor apparatus (GE Healthcare). After IEF, individual strips were incubated in equilibration buffer (50 mM Tris-HCl, 6 M urea, 30% glycerol, 2% SDS) supplemented with 1% DTT. This step was repeated using the same buffer with 4% iodoacetamide in place of 1% DTT. The proteins were then resolved in 12.5% SDS-PAGE gels using the Hoefer SE 600 Ruby apparatus (GE Healthcare) at 15 mA for 15 min and then at 30 mA at 20°C, until the bromophenol blue dye front had run off the bottom of the gel. To facilitate MS analysis, 500 µg of the unlabeled pooled protein sample for each group was run in parallel on a preparative gel and was stained using Deep Purple staining (GE Healthcare) according to the manufacturer's instructions.

### Gel image acquisition and analysis

The gel images for analysis were obtained by using the Typhoon 9700 Imager (Amersham Bioscience) and were processed in DeCyder 6.5 differential analysis software (Amersham Bioscience). The spots on the gels were codetected automatically as 2D DIGE image pairs, which intrinsically links a sample to its in-gel standard. Matching between gels was performed utilizing the in-gel standard from each image pair. The experimental setup and relationship between samples were assigned in DeCyder software. Each individual Cy3 or Cy5 gel image was assigned an experimental condition, and all Cy2 images were classified as standards. The gel with the highest spot count was considered the master gel. Statistical analysis was carried out for every matched-spot set, comparing the average and standard deviation of protein abundance for each spot between HuN4 and HuN4-F112 or between HuN4-F112 and PAM using Student's t-test, and the comparison among the three groups was carried out using ANOVA. The procedure was performed using the DeCyder DIA (Difference In-gel Analysis) and the DeCyder Biological Variation Analysisb (BVA) software module. Protein spots with significant differences in abundance (more than 1.2-fold, *P*<0.01) were selected in the stained preparative gels for spot selection.

### Mass spectrometry (MS) identification

Spots of interest from the preparative gels were manually excised. The gel samples were placed in a tube and were washed twice with 500 µL and 250 µL ddH_2_O for 15 min. For trypsin digestion, the gel samples were washed twice with 50 mM w/v NH_4_HCO_3_ and covered with 0.7 µL Porcine Trypsin solution (Promega, Madison, WI, USA) in 50 mM w/v NH_4_HCO_3_. After incubation overnight at 37°C, the supernatant was transferred to a second tube and 40 µL 50 mM w/v NH_4_HCO_3_ was added. Gel samples were washed with 40 µL of 50 mM w/v NH_4_HCO_3_, the supernatant was collected, and both collected supernatants were combined. Then, the collected solution was washed with 70% v/v ACN and dried in a Speed Vac (Vacuum Concentrator, Bachhofer). The peptide mixtures were desalted using ZipTip C-18 RP tips (Millipore, Billerica, MA, USA) that were moistened with 100% ACN and equilibrated with 0.1% TFA. Peptide samples, which were redissolved in 10 mL 0.5% TFA, were eluted with 50% ACN/0.1% TFA and were then dried in a Speed Vac (vacuum concentrator).

The purified peptides were spotted on a MALDI plate and covered with 0.7 mL of 2 mg/mL 3,5-Dimethoxy-4-hydroxycinnamic acid matrix (Sigma) with 10 mM NH_4_H_2_PO_4_ in 60% ACN. All samples were then analyzed by MALDI-TOF/TOF MS with a 4800 Proteomics Analyzer (Applied Biosystems, Foster City, CA). Monoisotopic peak masses were acquired in a mass range of 800–4,000 Da, with a signal-to-noise ratio (S/N) 200. Five of the most intense ion signals, excluding common trypsin autolysis peaks and matrix ion signals, were selected as precursors for MS/MS acquisition. The peptide mass fingerprint (PMF) combined MS/MS data were submitted to MASCOT version 2.1 (Matrix Science) for identification according to the SwissProt Sus scrofa database. The searching parameters were set as follows: Sus scrofa, trypsin cleavage (one missed cleavage permitted), fixed modifications, methionine oxidation as a variable modification, peptide mass tolerance set at 100 ppm, and fragment tolerance set at 0.8 Da. The required criteria for the successful identification of a protein were as follows: an ion score confidence interval (C.I.%) for PMF and MS/MS data ≥95%, and a peptide count (hit) ≥4. At least two peptides with distinct sequences were identified in the MS/MS analysis.

### Gene annotation and functional classification

Gene Ontology (GO) annotation and functional classification of the identified proteins was performed with Blast2GO [24], [25], the version is V2.6.2 and the current public database is b2g_aug12 (www.blast2go.com). A nonredundant database was used as reference for Blastp searches with an expectation value minimum of 1×e^−3^ and a high scoring segment pair cut-off of 33. Annotations were made with default parameters. Briefly, the pre-eValue-Hit-Filter was 1×e^−6^, the Annotation cut-off was 55, and the GO Weight was 5. The percentages of assigned GOs in level 2 of biological process, molecular function and cellular component were calculated.

### Construction of protein-protein interaction networks

We performed a protein-protein interaction network analysis on the identified differentially expressed proteins. We utilized String software (http://string-db.org/) to search the String database and the protein-protein interaction network database and subsequently built a protein-protein interaction network for all of the differentially expressed proteins.

### Validation of differentially expressed proteins via real-time PCR

The virulent HuN4 strain and the attenuated HuN4-F112 strain were used to infect PAM cells separately. Infected cells were harvested 24 hours and 48 hours after infection, followed by extraction of total cellular RNA in accordance with the instructions of the RNeasy Plus Mini Kit (Qiagen). The RNA concentration and purity were determined based on the A260 and A280 values, and the RNA concentration was adjusted to 250 ng/µL. Real-time PCR was performed using a kit from Fermentas Inc. according to the manufacturer's instructions. Total RNA was reverse transcribed into cDNA. PCR was performed in a reaction volume of 25 µL containing 12.5 µL of Maxima SYBR Green ROX qPCR Master Mix (2×), 0.5 µL of each of the primers (10 µM) (Table 2), and 1 µL of cDNA. The reaction conditions were as follows: 50°C for 2 minutes, 95°C for 10 minutes, and 40 cycles of 95°C for 15 seconds→65°C for 60 seconds (for fluorescence detection). Additionally, a melting curve analysis was performed at 65°C to 95°C at the end of the reaction. All samples were used in three independently repeated experiments, and the average value obtained from the three experiments was taken as the quantitative result. Quantitative analysis was performed using the quantitation-comparative C_T_ (ΔΔC_T_) mode in ABI 7500 software, version 2.0.1 (USA). The uninfected group was used for calibration (relative expression = 1), and β-actin was used as the internal reference gene. Finally, a single factor analysis of variance (ANOVA) was used to analyze the data statistically, with *P*<0.05 indicating statistically significant differences and *P*<0.01 indicating highly statistically significant differences.

**Table 2:** Real-time RT-PCR primer sequences and amplicon lengths of differentially expressed proteins.

Genes	Accession	Primer	Sequences (5′-3′)	Length
PKM2	XM_001929069	PKM2-U	CTGAGGGCAGTGATGTGGC	200 bp
		PKM2-L	GGTAGGGTCGCTGGTAATGG	
HSPB1	XM_003354494	HSPB1-U	CCAAGGACGGCGTGGTGGAGAT	222 bp
		HSPB1-L	CCTCGAAAGTGACAGGGATGGTGA	
PSMA6	FJ358606	PSMA6-U	TCCCAGGTACAGAGGGCACG	150 bp
		PSMA6-L	CATACAACAACCAAGAGGC	
β-actin	NM_001101	Actin-U	TCATCACCATTGGCAATGAG	157 bp
		Actin-L	AGCACTGTGTTGGCGTACAG	

Transcript levels were normalized relative to those of β-actin.

## Results

### 2D-DIGE screening of differentially expressed protein spots

**Figure 1:**
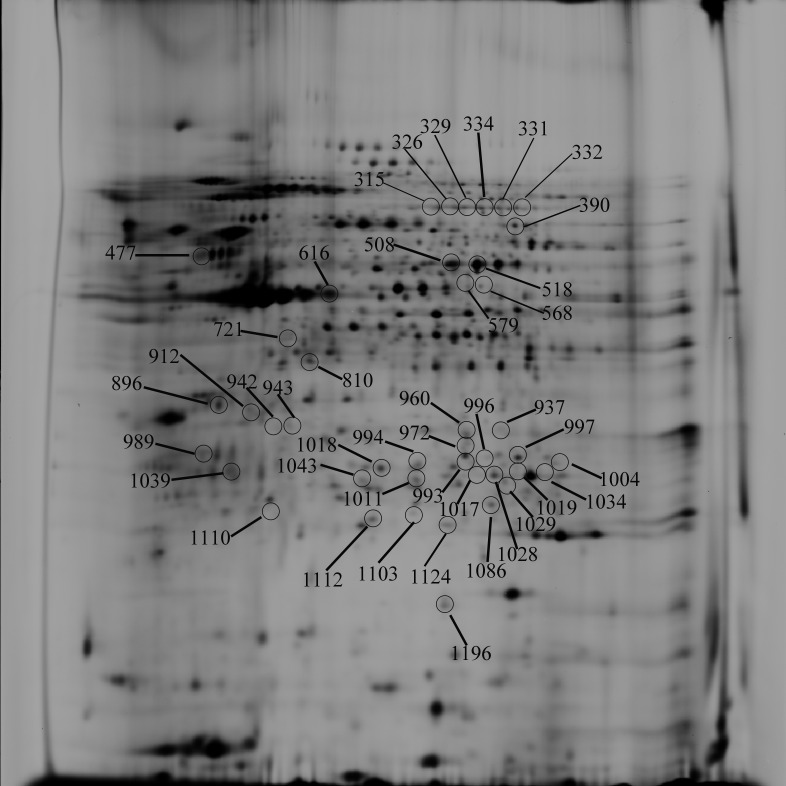
2D-DIGE analysis of PRRSV-infected PAMs and mock-infected PAMs. Arrows indicate isolated and identified protein spots that were up- or downregulated by at least 1.2-fold (*P*<0.01). Spots are numbered according to Table 3. Equal amounts of total protein from infected and uninfected whole cell lysates were resolved by 2D-DIGE.

The reproducibility of the system was evaluated by running an identical sample three times. Figure 1A shows a representative 2D-DIGE image. Approximately, a total of 1492 Protein spots were resolved in a single master gel image of this reproducibility experiment. Image analysis revealed a total of 153 differentially expressed protein spots in the PAMs infected with the virulent HuN4 strain or the attenuated HuN4-F112 strain (with an average ratio >1.2 or <−1.2, *P*<0.01) (Table 3). 73, 70, and 98 protein spots differentially expressed between different conditions (HuN4 vs PAM, HuN4-F112 vs PAM, and HuN4-F112 vs HuN4) (Table S1, S2, S3).

**Table 3:** Statistical analysis of differentially expressed proteins. Image analysis detected a total of 153 differentially expressed protein spots in the PAMs infected with the virulent HuN4 strain or the attenuated HuN4-F112 strain (with an average ratio >1.2 or <−1.2, *P*<0.01).

Master No.	1-ANOVA	Master No.	1-ANOVA	Master No.	1-ANOVA
691	5.30E-09	815	0.00032	1060	0.0023
997	7.50E-07	912	0.00041	1096	0.0024
1033	1.40E-06	960	0.00045	1030	0.0024
329	1.50E-06	255	0.00045	288	0.0024
993	1.60E-06	1179	0.00046	1222	0.0025
996	2.10E-06	1196	0.00049	999	0.0027
142	3.70E-06	969	0.00054	515	0.0028
334	3.80E-06	972	0.00057	1149	0.003
944	4.90E-06	839	0.00061	275	0.0031
1010	6.10E-06	886	0.00068	1163	0.0032
721	8.10E-06	661	0.00068	670	0.0035
1142	9.30E-06	937	0.00071	524	0.0038
1143	1.40E-05	508	0.00071	461	0.004
889	1.40E-05	967	0.00073	525	0.0041
1028	1.50E-05	1019	0.00074	1073	0.0042
1017	1.60E-05	684	0.00077	678	0.0042
331	1.70E-05	896	0.00078	522	0.0045
1144	2.50E-05	810	0.00078	1115	0.0048
1011	2.90E-05	616	0.00081	990	0.005
326	3.40E-05	579	0.00085	818	0.0051
942	4.00E-05	483	0.00088	1139	0.0052
245	4.40E-05	390	0.00088	612	0.0056
1018	5.60E-05	1031	0.00089	802	0.0057
1100	6.60E-05	1112	0.00091	785	0.0058
518	7.00E-05	1039	0.00094	274	0.0058
591	7.20E-05	602	0.00095	516	0.006
573	7.80E-05	796	0.001	1109	0.0061
568	8.10E-05	778	0.001	138	0.0062
682	8.40E-05	1165	0.0012	232	0.0063
1029	8.60E-05	1041	0.0012	1120	0.0064
332	9.30E-05	910	0.0012	692	0.0065
1140	9.60E-05	662	0.0012	646	0.0067
943	0.00012	1176	0.0013	386	0.0067
941	0.00012	1094	0.0013	1071	0.0068
1124	0.00013	781	0.0013	269	0.0069
1103	0.00013	482	0.0013	1125	0.0072
1086	0.00013	263	0.0013	970	0.0072
826	0.00014	1114	0.0015	330	0.0073
989	0.00016	665	0.0015	1076	0.0079
882	0.00019	534	0.0015	622	0.0081
854	0.00022	366	0.0015	521	0.0081
572	0.00022	977	0.0016	888	0.0082
477	0.00024	774	0.0016	660	0.0084
1004	0.00026	1183	0.0018	599	0.0085
994	0.00026	1084	0.0018	980	0.0086
657	0.00027	578	0.0018	728	0.0086
683	0.00028	530	0.0019	924	0.009
823	0.00029	420	0.0019	653	0.0091
315	0.00029	932	0.002	673	0.0094
1043	0.0003	619	0.002	1221	0.0098
1034	0.00032	567	0.002	592	0.0098

### MALDI-TOF/TOF MS identification of differentially expressed proteins

MALDI-TOF/TOF MS was used to analyze the samples and the protein identity and partial sequence information was obtained by GPS Explorer and MASCOT software. We picked and analyzed 42 spots of the total 153 spots, 27 of which were successfully identified as 24 proteins (Table 4). Spots 1017 and 1028 were identified as triosephosphate isomerase 1. Spots 1043, 1039 and 993 were identified as heat shock protein beta-1(heat shock protein 27 kDa). Spots 616 and 1034 were identified as keratin 18.

**Table 4:** Identification of differentially expressed proteins in porcine alveolar macrophages (PAMs) infected with HuN4 and HuN4-F112 by MALDI-TOF/TOF.

Master no.^a^	Accession no.^b^	Porcine protein name	Protein score^c^	Theoretical MW(Da)^d^/pI^e^	Experimental MW(kDa)/pI	Peptide count^f^	coverage (%)^g^	Protein score C.I.%^h^	Fold change^i^		
									HuN4/PAM	HuN4-F112/PAM	HuN4-F112/HuN4
**332**	gi|194038726	pyruvate kinase 3 isoform 2	406	58411.3/7.96	60/7.3	21	41%	100	−2.02	−1.42	1.42
**390**	gi|166977567	D-3-phosphoglycerate dehydrogenase, Short = 3-PGDH	248	57514.8/6.44	57/7.4	12	24%	100	−1.44	1.03	1.47
**477**	gi|194041761	similar to sorbin and SH3 domain containing 1	60	146416.6/8.92	50/5.0	17	22%	97.806	−1.7	1.06	1.81
**616**	gi|157382506	keratin 18	186	18038.2/4.63	42/5.9	5	35%	100	1.37	−1.33	−1.82
**568**	gi|262204914	phosphoglycerate kinase 1	229	35595.6/8.94	43/7.1	7	33%	100	−1.15	1.3	1.5
**579**	gi|160858224	CArG-binding factor A	154	32055.7/8.31	37/7.0	7	21%	100	2.01	1.17	−1.72
**810**	gi|75045190	Full = Tubulin beta chain, AltName: Full = Tubulin beta-5	79	50095.1/4.78	30/5.9	7	23%	99.969	1.74	1.04	−1.68
**942**	gi|194042750	similar to pyrophosphatase 1	224	44205.2/8.81	30/5.6	7	28%	100	−2.62	−1.04	2.52
**912**	gi|148613357	F-actin capping protein alpha 1 subunit	132	33167.4/5.53	32/5.4	3	15%	100	−1.61	−1.13	1.42
**943**	gi|114152157	Serine/arginine-rich splicing factor 1	548	27841.9/10.37	30/5.8	19	56%	100	−1.3	1.02	1.32
**896**	gi|45269029	cytoskeletal beta actin	93	45162.4/5.55	32/5.1	6	29%	100	1.28	−1.45	−1.86
**989**	gi|194036973	14-3-3 protein zeta/delta	281	27898.8/4.73	28/5.3	14	48%	100	−1.07	1.1	1.18
**1039**	gi|55926209	heat shock protein beta-1	90	22984.7/6.23	27/5.3	3	40%	100	−1.57	−1.04	1.51
**1043**	gi|50916342	heat shock protein 27kDa	111	14268.2/5.94	27/6.2	3	8%	99.983	−1.1	1.4	1.55
**1018**	gi|194043069	similar to Splicing factor, arginine/serine rich 9	131	25672.4/8.74	28/6.3	11	27%	100	−1.54	1.14	1.76
**994**	gi|194043043	endoplasmic reticulum resident protein 29	75	29325.3/6.85	28/6.8	5	16%	99.934	−1.73	1.19	2.06
**993**	gi|55926209	heat shock protein beta-1	130	22984.7/6.23	28/7.0	3	20%	99.95	−1.41	1.38	1.94
**960**	gi|194042917	similar to 26S proteasome non-ATPase regulatory subunit 9	133	40435/4.82	30/7.0	4	24%	100	−1.61	−1.39	1.15
**937**	gi|160419232	Proteasome subunit beta type-7	86	30303.3/6.9	30/7.1	3	10%	99.994	−1.36	−2.07	−1.52
**1017**	gi|262263205	triosephosphate isomerase 1	165	26878.9/6.54	28/7.0	11	58%	100	−2.05	−1.39	1.47
**1028**	gi|262263205	triosephosphate isomerase 1	360	26878.9/6.54	27/7.1	13	61%	100	−1.02	1.7	1.73
**997**	gi|210062872	proteasome subunit alpha type 6	317	27884/6.34	28/7.4	11	43%	100	−1.23	1.43	1.75
**1112**	gi|1717797	PRDX2	125	14272.2/4.7	25/6.3	3	15%	100	3.44	1.97	−1.75
**1103**	gi|67038668	DJ-1 protein	110	20094.6/6.33	25/6.6	5	34%	100	−1.24	1.16	1.44
**1196**	gi|195562242	actin related protein 2/3 complex subunit 5-like protein	244	16944.8/6.15	20/6.9	5	51%	100	−1.16	−3.13	−2.7
**1034**	gi|157382506	keratin 18	191	18038.2/4.63	27/7.5	6	33%	100	−1.11	1.74	1.93
**1086**	gi|222136592	proteasome subunit beta type-3	421	23277.6/5.76	26/7.1	8	36%	100	2.72	1.68	−1.62

a Protein spot numbers on 2-DE gel refers to the protein spot labels shown in Fig. 1.

b Accession number obtained through the input of MALDI-TOF MS/MS experimental results in a MASCOT search of the NCBI nr database.

c MASCOT protein score based on combined MS and MS/MS spectra >60 (*P*≤0.05).

d Theoretical molecular mass.

e Theoretical pI.

f Observed peptides that differ either by sequence, modification or charge.

g .Sequence coverage is based on peptides with a unique sequence.

h protein score confidence interval %.

i Fold change is calculated by DeCyder software (Version 6.5).

### GO annotation and functional classification

**Figure 2:**
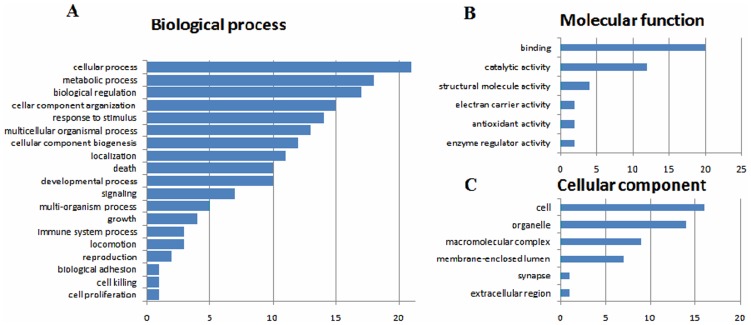
Functional classification. Column diagrams showing the gene ontology (GO) distribution of differentially expressed proteins according to major biological process categories (A), molecular function categories (B), and cellular component categories (C). The Y axis represents the percentage (%) of GO terms.

Gene Ontology annotation was performed for the 24 differentially expressed proteins. First, the Blast2GO was used to download annotated porcine protein data from the NCBI non-redundant database, and GO functional classification was performed for every annotated known protein. In the present study, 24 single sequences were successfully matched with one or more GO phrases, and these 256 matched GO phrases were functionally classified into three unrelated GO functions (GO ontologies): biological processes, molecular functions, and cellular components (Figure 2). Among these categories, biological processes accounted for 145 sequences (56.7%), molecular functions for 38 sequences (14.8%), and cellular components for 73 sequences (28.5%). The biological processes class involved the following four major categories: cell processes, metabolism, biological regulation, and cell structural components. This class was also associated with the functions immune regulation and signal transduction, including six sequences (4%) involved in immune system processes, eight sequences (6%) involved in signaling pathways, and eleven sequences (8%) involved in response to stimulus. In the molecular functions class, 18 sequences (47%) presented the function of binding with other molecules, and 12 sequences (31%) had catalytic functions.

### Analysis of protein-protein interactions

**Figure 3:**
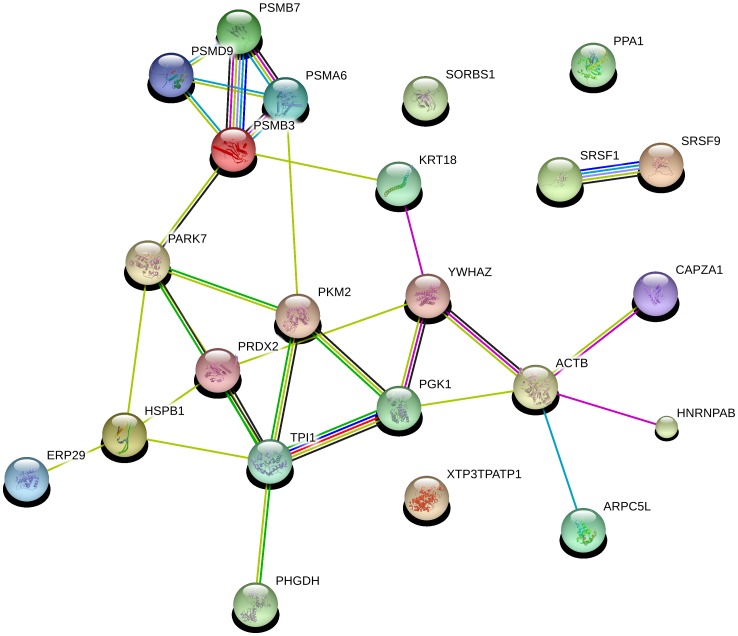
The protein-protein interaction network as analyzed by String software. An edge was drawn with up to seven differently colored lines that represent the existence of the seven types of evidence used in predicting the associations. A red line indicates the presence of fusion evidence; a green line indicates neighborhood evidence; a blue line indicates co-occurrence evidence; a purple line indicates experimental evidence; a yellow line indicates text-mining evidence; a light blue line indicates database evidence; and a black line indicates coexpression evidence.

String software was used to search the String database and the protein-protein interaction network database, and a protein interaction network diagram was constructed for the 24 differentially expressed proteins. The proteins PKM2 (pyruvate kinase isozyme M2), YWHAZ (tyrosine 3-monooxygenase/tryptophan 5-monooxygenase activation protein, zeta polypeptide), and PRDX2 (peroxiredoxin 2) were located in the most central area of the network, followed by PSMA6 (proteasome subunit alpha type 6), KRT18 (keratin 18), ACTB (beta-actin), PGK1 (phosphoglycerate kinase 1), TPI1 (triosephosphate isomerase 1), HSPB1 (heat shock protein beta-1), PARK7 (Parkinson disease (autosomal recessive, early onset) 7), and PSMB3 (proteasome subunit beta type 3). ERP29 (endoplasmic reticulum protein 29), PHGDH (phosphoglycerate dehydrogenase), HNRNPAB (heterogeneous nuclear ribonucleoprotein A/B), CAPZA1 (capping protein (actin filament) muscle Z-line, alpha 1), and ARPC5L (actin related protein 2/3 complex, subunit 5-like) were located in the outermost part of the network, and some differentially expressed proteins were not included in the network (Figure 3).

### Real-time PCR analysis of differentially expressed genes

**Figure 4:**
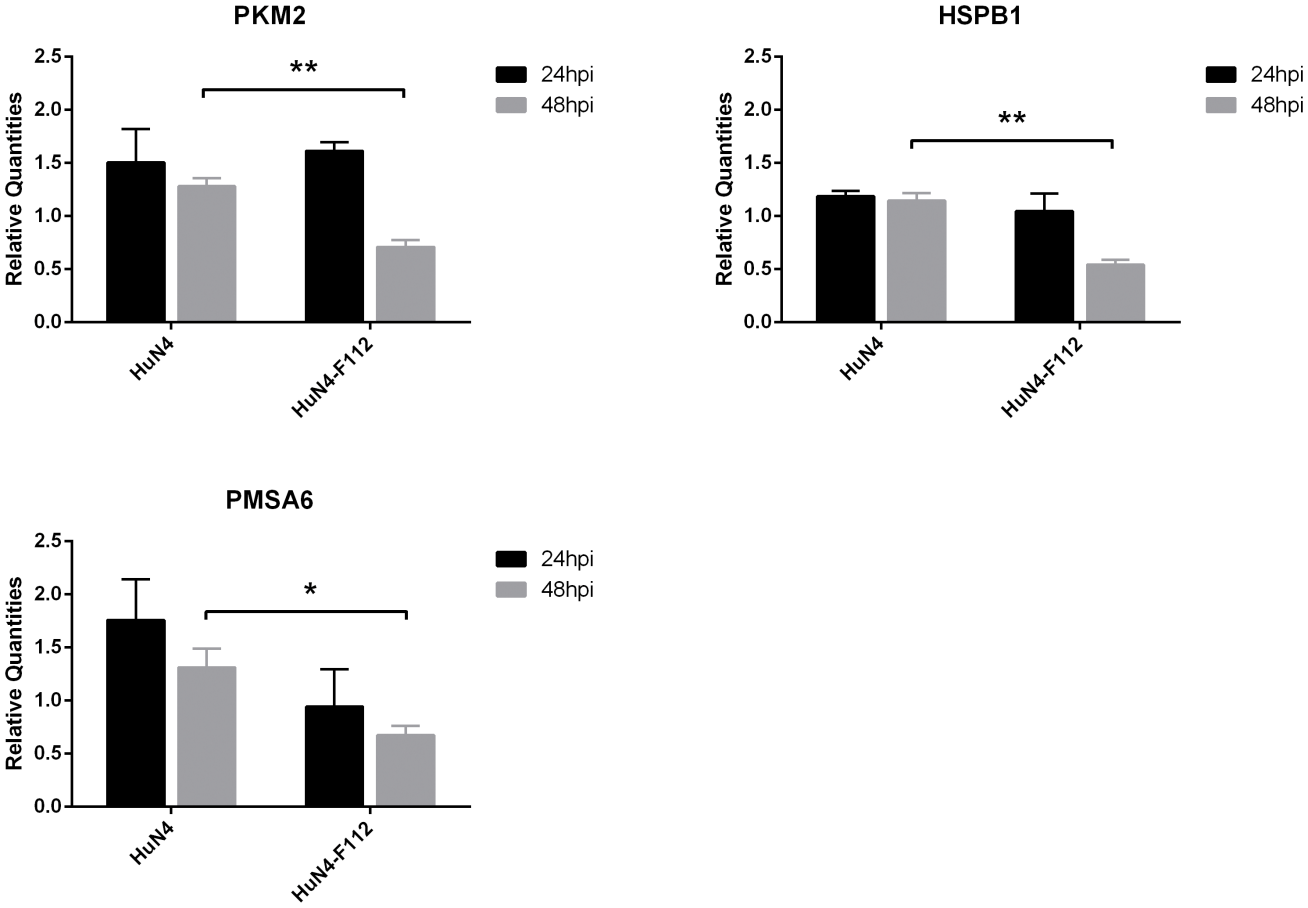
Confirmation of the transcriptional regulation of DEPs by real-time RT-PCR. Transcript alteration of three selected genes in PAM cells from the PRRSV-infected group compared with the mock-infected group. Total RNA extracted from PAM cells was measured by real-time RT-PCR analysis; relative expression levels were calculated according to the 2^ΔΔ^CT method, using β-actin as an internal reference gene and the mock-infected group as a calibrator (relative expression = 1). Error bars represent the standard deviation. Please refer to Table 2 for the identification of gene symbols that represent different genes.

Real-time PCR assay was done to confirm some differences found by 2D-DIGE and MS in the current study. The result, PKM2 was upregulated at 24 hpi by infection with both the HuN4 strain and the HuN4-F112 strain. However, it is interesting that at 48 hpi PKM2 was still upregulated in cells with HuN4 infection but downregulated in cells with HuN4-F112, and the difference is significant (*P*<0.01). The expression of HSPB1 was upregulated upon infection with the HuN4 strain and downregulated upon infection with the HuN4-F112 strain, and the differences between cells infected with HuN4 and HuN4-F112 strains were significant at 48 hpi (*P*<0.01). Similar to HSPB1, PSMA6 was also upregulated with HuN4 infection and downregulated when infected with HuN4-F112, and the difference at 48 hpi between them was significant (*P*<0.05), too. So, all the differences in Real-time PCR assay confirmed those we found by 2D-DIGE and MS(Figure 4).

## Discussion

There were significant differences between the virulent HP-PRRSV strain, HuN4, and its attenuated form, HuN4-F112, detected in our *in vivo* pathogenicity tests [5], [22]. Focusing on the causes of the differences in pathogenicity between the two strains, we previously analyzed the strains at the level of viral genes and revealed that there were only 41 amino acid differences between the two strains [23]. Two infectious molecular clones of virulent HuN4 and HuN-F112 strains were created, and several chimeric strains by mixing those two were also made, to study viral pathogenicity. However, we have not yet identified any amino acid differences being responsible for the different pathogenicity between HuN4 and HuN-F112 [26], and we are still working on it. On the other hand, we also attempted to identify relevant factors underlying the different responses to infection with HuN4 and virulent HuN4-F112 strains within host PAM cells, so in the present study, we did 2D-DIGE followed by MS to screen differential protein expression after infecting of PAM cells with HuN4 and HuN-F112 strains. Knowing protein level changing caused by viral infection may be able to allow us to better understand the mechanism of viral pathogenicity, too.

PAM cells infected with the virulent HuN4 strain and the attenuated HuN4-F112 strain were collected for utilizing fluorescent two-dimensional difference gel electrophoresis (2D-DIGE) to screen for intracellular proteins that were differentially expressed in host cells infected with the two strains. There were 153 proteins with significant different expression (*P*<0.01) observed, 42 of which were subjected to mass spectrometry. Among those 42 samples, in this study, we identified 24 differentially expressed proteins that might be involved to varying degrees and could play different roles in the processes leading to the differences between the two strains. The validation of a number of differentially expressed proteins showed that the expression of PKM2, HSPB1, and PSMA6 was significantly different during the infection course between the virulent HuN4 strain and the attenuated HuN4-F112 strain.

Different viruses have different methods for regulating cell metabolism by regulating certain cellular protein level or concentration of particular lipids, like Hepatitis C virus [27], hepatitis B virus [28], human cytomegalovirus (HCMV) and herpes simplex virus 1 (HSV-1) [29]. Viruses may first regulate enzymes involved in cellular metabolic pathways to utilize the cellular metabolism of energy and substances for viral synthesis [29]–[31]. Here, we found that PKM2 was upregulated when PAMs were infected with the HuN4 strain and downregulated when PAMs were infected with the HuN4-F112 strain. PKM2 is an enzyme that participates in the metabolism of carbohydrates and is mainly involved in glycolysis. It has also been observed that when an oncogenic virus causes tumor, glycolysis is enhanced in the tumor cells, and that it is glycolysis, not aerobic metabolism, provides the energy supply (the mitochondrial oxidative phosphorylation reaction is inhibited). Although less ATP is produced per monosaccharide molecule during glycolysis than during aerobic metabolism, increased glycolysis and reduced oxidative phosphorylation could increase the production of ATP without generating reactive oxygen species, which was known as Warburg effect [32], [33]. The Warburg effect is required for the infectivity of many viruses, such as the Kaposi's sarcoma herpesvirus [33], feline leukemia virus [34], Rous sarcoma virus [35], cytomegalovirus [36], and white spot syndrome virus [37]. The Warburg effect facilitates viral replication and long-term viral existence in cells [32]. Here, potential role of PKM2 in PRRSV infection needs to be addressed by further study.

Our study showed that HSPB1 in PAM cells was up- and down-regulated by the virulent HuN4 strain and the attenuated HuN4-F112 strain, respectively, which confirmed related previous studies [18], [20]. In addition, infections involving many other viruses, such as the avian influenza H9N2 [38], the Epstein-Barr virus [39], the African swine fever virus [40], IBDV [41] and CSFV [42] also induce upregulation of HSP27 expression. HSPB1, also known as heat shock protein 27 (HSP27), belongs to the small heat shock protein (HSP) family and is a ubiquitous protein induced by stress. To release large quantities of virus particles during the virus release phase, the virus delays cellular apoptosis through different strategies in the early stages of the viral infection of cells to provide sufficient time for viral replication. Hsp27 regulates apoptosis through an ability to interact with key components of the apoptotic signaling pathway, in particular, those involved in caspase activation and apoptosis [43]. HSPB1 is also involved in the cellular protective responses to various stresses, such as heat shock, toxins, and oxidative stress, as well as stress-induced HSP regulation, the mitogen-activated protein kinase (MAPK) signaling pathway, and the regulation of translation initiation, molecular chaperones, actin organization, and cell motility, and, any change of which could affect PRRSV infection, but we still need to do more study to find out.

In the protein-protein network diagram, PSMA6, PSMB3, PSMD9 (proteasome (prosome, macropain) 26S subunit, non-ATPase, 9), and PSMB7 (proteasome (prosome, macropain) subunit, beta type 7) all belong to the ubiquitin-proteasome pathway (UPP), and all of these proteins showed significant changes between the PAM cells infected with the virulent strain and the cells infected with the attenuated strain. PSMA6 was upregulated following infection with the HuN4 strain in the present study and was downregulated by infection with the HuN4-F112 strain. The UPP is the major intracellular degradation system that performs degradation of foreign proteins and is the system that a virus must avoid to evade immune surveillance and achieve virus particle maturation, release, replication, and activation from the latent state [44], [45]. It has been reported that many viruses employ unique approaches for co-opting the UPP pathway for the replication of their viral particles [44]. Activation of the UPP pathway is necessary for hepatitis E virus replication [46] and is also required by other viruses, such as the hepatitis B virus [47], [48], influenza A virus [49], vaccinia virus [50], herpes simplex virus [51], and rotavirus [52]. Zhang et al. [18] studied the infection of PAMs by HP-PRRSV using proteomic methods and found that HP-PRRSV upregulated the expression of UPP-related proteins, suggesting that these proteins might enhance HP-PRRSV replication in PAMs. Our results also showed that the virulent HuN4 strain upregulated the expression of UPP-related proteins, while the attenuated HuN4-F112 strain downregulated the expression of UPP-related proteins, suggesting that the virulent PRRSV strain might utilize the UPP pathway to promote viral replication. However, determination of whether PRRSV employs a similar approach to evade host immune surveillance and whether the virulent and attenuated strains differ in their ability to regulate and utilize the UPP pathway, thus leading to their different replication capacities in cells, still requires further experimental validation.

In summary, we utilized proteomic approaches to identify differential expression of cellular proteins between PAM cells infected with either the virulent HuN4 strain or the attenuated HuN4-F112 vaccine strain. Functional analysis of the proteins showed that the differentially expressed proteins were correlated with the degree of infectivity of the virulent/attenuated strains in the target PAM cells and suggested that these differentially expressed proteins are involved in the pathogenic mechanisms of PRRSV strains with different pathogenicities. Our findings provide new information that can be used in future studies addressing the differences in pathogenicity between PRRSV virulent and attenuated strains.

## Supporting Information

Table S1 **The differential expressed protein spots between HuN4 and PAM(with an average ratio >1.2 or <−1.2,** ***P*** **<0.01).**
(DOC)

Table S2 **The differential expressed protein spots between HuN4-F112 and PAM (with an average ratio >1.2 or <−1.2,** ***P*** **<0.01).**
(DOC)

Table S3 **The differential expressed protein spots between HuN4-F112 and HuN4 (with an average ratio >1.2 or <−1.2,** ***P*** **<0.01).**
(DOC)
