# Supplementary material for: Identification of Differentially Expressed Proteins in Porcine Alveolar Macrophages Infected with Virulent/Attenuated Strains of Porcine Reproductive and Respiratory Syndrome Virus
Source: PLoS One. 2014 Jan 21;9(1):e85767. doi: 10.1371/journal.pone.0085767 (PMC3897507; doi:10.1371/journal.pone.0085767)
Supplement: Table S1 — The differential expressed protein spots between HuN4 and PAM(with an average ratio >1.2 or <−1.2, P <0.01). (DOC) [file pone.0085767.s001.doc]

| **Master No.** | **T-test** | **Av. Ratio** |
| --- | --- | --- |
| **691** | 5.43E-06 | 15.84 |
| **572** | 6.04E-03 | 15.22 |
| **823** | 1.85E-03 | 6.63 |
| **602** | 6.68E-03 | 6.23 |
| **573** | 2.81E-03 | 5.63 |
| **684** | 8.67E-03 | 4.56 |
| **854** | 2.57E-03 | 3.94 |
| **683** | 5.00E-03 | 3.77 |
| **1165** | 4.43E-03 | 3.73 |
| **826** | 2.50E-03 | 3.67 |
| **1112** | 3.13E-03 | 3.44 |
| **664** | 6.54E-03 | 3.41 |
| **1143** | 7.95E-04 | 2.91 |
| **660** | 9.54E-03 | 2.85 |
| **1163** | 5.26E-03 | 2.83 |
| **815** | 1.75E-03 | 2.75 |
| **1086** | 2.05E-03 | 2.72 |
| **1096** | 8.47E-04 | 2.41 |
| **1142** | 7.45E-04 | 2.33 |
| **882** | 4.18E-03 | 2.18 |
| **889** | 2.34E-03 | 2.02 |
| **579** | 8.22E-03 | 2.01 |
| **1144** | 6.04E-03 | 1.91 |
| **1140** | 8.52E-03 | 1.8 |
| **810** | 3.37E-03 | 1.74 |
| **245** | 3.52E-03 | 1.74 |
| **1076** | 8.01E-03 | 1.71 |
| **1120** | 6.88E-03 | 1.66 |
| **665** | 8.93E-03 | 1.47 |
| **687** | 2.37E-03 | 1.27 |
| **619** | 6.02E-03 | 1.21 |
| **386** | 2.62E-03 | -1.29 |
| **1051** | 2.53E-03 | -1.3 |
| **943** | 3.11E-03 | -1.3 |
| **922** | 4.05E-03 | -1.32 |
| **1004** | 2.94E-03 | -1.35 |
| **993** | 2.41E-03 | -1.41 |
| **1037** | 6.51E-03 | -1.42 |
| **390** | 1.15E-03 | -1.44 |
| **972** | 8.41E-03 | -1.46 |
| **1018** | 3.74E-03 | -1.54 |
| **796** | 2.30E-03 | -1.58 |
| **186** | 3.39E-03 | -1.6 |
| **960** | 2.66E-03 | -1.61 |
| **912** | 3.47E-03 | -1.61 |
| **525** | 4.94E-03 | -1.65 |
| **781** | 7.26E-03 | -1.66 |
| **941** | 8.80E-04 | -1.67 |
| **1491** | 8.16E-03 | -1.71 |
| **994** | 7.20E-03 | -1.73 |
| **721** | 6.38E-03 | -1.79 |
| **932** | 7.09E-03 | -1.79 |
| **318** | 7.04E-04 | -1.8 |
| **199** | 1.94E-03 | -1.85 |
| **774** | 8.47E-03 | -1.85 |
| **331** | 7.55E-04 | -1.87 |
| **1100** | 7.24E-03 | -1.89 |
| **315** | 4.51E-03 | -1.93 |
| **142** | 2.91E-03 | -2 |
| **332** | 2.33E-03 | -2.02 |
| **208** | 6.19E-03 | -2.02 |
| **334** | 2.68E-04 | -2.03 |
| **420** | 8.15E-03 | -2.03 |
| **1017** | 5.57E-04 | -2.05 |
| **212** | 3.14E-05 | -2.07 |
| **483** | 7.32E-03 | -2.09 |
| **329** | 2.83E-04 | -2.14 |
| **326** | 1.47E-03 | -2.17 |
| **944** | 2.30E-04 | -2.38 |
| **1011** | 9.15E-04 | -2.38 |
| **1061** | 2.56E-03 | -2.46 |
| **942** | 9.44E-04 | -2.62 |
| **1010** | 2.43E-04 | -3.02 |
