# Supplementary material for: Identification of Differentially Expressed Proteins in Porcine Alveolar Macrophages Infected with Virulent/Attenuated Strains of Porcine Reproductive and Respiratory Syndrome Virus
Source: PLoS One. 2014 Jan 21;9(1):e85767. doi: 10.1371/journal.pone.0085767 (PMC3897507; doi:10.1371/journal.pone.0085767)
Supplement: Table S2 — The differential expressed protein spots between HuN4-F112 and PAM (with an average ratio >1.2 or <−1.2, P <0.01). (DOC) [file pone.0085767.s002.doc]

| **Master No.** | **T-test** | **Av. Ratio** |
| --- | --- | --- |
| **1196** | 1.87E-03 | 3.13 |
| **522** | 6.93E-03 | 2.56 |
| **482** | 2.96E-03 | 2.28 |
| **205** | 2.35E-03 | 2.24 |
| **937** | 2.46E-03 | 2.07 |
| **781** | 8.21E-03 | 2.03 |
| **999** | 3.52E-03 | 1.93 |
| **785** | 3.11E-03 | 1.82 |
| **1010** | 5.90E-04 | 1.8 |
| **910** | 5.53E-03 | 1.78 |
| **941** | 1.06E-03 | 1.76 |
| **330** | 1.60E-03 | 1.6 |
| **631** | 1.34E-03 | 1.49 |
| **932** | 9.80E-03 | 1.49 |
| **936** | 9.30E-03 | 1.46 |
| **326** | 1.58E-03 | 1.44 |
| **1011** | 3.99E-03 | 1.44 |
| **332** | 1.13E-03 | 1.42 |
| **315** | 4.08E-03 | 1.42 |
| **310** | 6.88E-03 | 1.4 |
| **331** | 5.94E-05 | 1.39 |
| **329** | 7.78E-04 | 1.39 |
| **960** | 5.33E-03 | 1.39 |
| **1017** | 9.50E-03 | 1.39 |
| **334** | 1.11E-03 | 1.38 |
| **944** | 5.81E-03 | 1.37 |
| **263** | 9.98E-03 | 1.34 |
| **1176** | 9.09E-03 | 1.31 |
| **622** | 7.28E-03 | 1.28 |
| **728** | 4.08E-03 | 1.26 |
| **302** | 5.84E-03 | 1.21 |
| **1015** | 8.43E-03 | -1.21 |
| **983** | 2.93E-03 | -1.22 |
| **1122** | 5.99E-03 | -1.29 |
| **568** | 2.90E-03 | -1.3 |
| **708** | 6.23E-03 | -1.31 |
| **1461** | 4.32E-03 | -1.36 |
| **993** | 4.49E-03 | -1.38 |
| **1043** | 4.55E-03 | -1.4 |
| **997** | 7.59E-04 | -1.43 |
| **996** | 6.63E-04 | -1.49 |
| **518** | 5.62E-04 | -1.52 |
| **1031** | 6.31E-03 | -1.54 |
| **659** | 8.46E-03 | -1.54 |
| **1300** | 3.34E-03 | -1.57 |
| **591** | 5.03E-04 | -1.6 |
| **578** | 7.41E-03 | -1.64 |
| **1033** | 5.73E-04 | -1.66 |
| **1028** | 1.25E-03 | -1.7 |
| **1114** | 4.82E-03 | -1.71 |
| **1034** | 3.18E-03 | -1.74 |
| **521** | 6.79E-03 | -1.76 |
| **1060** | 2.97E-03 | -1.79 |
| **1019** | 8.07E-03 | -1.79 |
| **1140** | 3.27E-03 | -1.83 |
| **1029** | 1.43E-03 | -1.87 |
| **839** | 2.84E-03 | -1.99 |
| **815** | 8.56E-03 | -2.03 |
| **664** | 3.96E-03 | -2.05 |
| **967** | 5.50E-04 | -2.09 |
| **969** | 1.64E-03 | -2.13 |
| **1142** | 2.86E-04 | -2.2 |
| **1144** | 6.19E-04 | -2.3 |
| **1143** | 2.45E-04 | -2.33 |
| **1094** | 2.88E-04 | -2.43 |
| **653** | 5.60E-04 | -2.45 |
| **1165** | 6.71E-03 | -2.89 |
| **823** | 9.65E-03 | -3.08 |
| **657** | 3.54E-03 | -7.66 |
| **691** | 9.38E-06 | -8.43 |
|  |  |  |

|  |
| --- |
