# Supplementary material for: Identification of Differentially Expressed Proteins in Porcine Alveolar Macrophages Infected with Virulent/Attenuated Strains of Porcine Reproductive and Respiratory Syndrome Virus
Source: PLoS One. 2014 Jan 21;9(1):e85767. doi: 10.1371/journal.pone.0085767 (PMC3897507; doi:10.1371/journal.pone.0085767)
Supplement: Table S3 — The differential expressed protein spots between HuN4-F112 and HuN4 (with an average ratio >1.2 or <−1.2, P <0.01). (DOC) [file pone.0085767.s003.doc]

| **Master No.** | **T-test** | **Av. Ratio** |
| --- | --- | --- |
| **1163** | 5.09E-03 | 4.51 |
| **612** | 9.11E-04 | 4.4 |
| **602** | 8.04E-03 | 3.82 |
| **889** | 1.70E-04 | 3.09 |
| **1179** | 1.03E-03 | 3.08 |
| **684** | 1.21E-04 | 2.81 |
| **1196** | 2.88E-03 | 2.7 |
| **682** | 1.97E-03 | 2.67 |
| **819** | 1.12E-03 | 2.55 |
| **661** | 2.70E-03 | 2.48 |
| **683** | 7.74E-03 | 2.34 |
| **43** | 2.18E-03 | 2.28 |
| **245** | 2.04E-03 | 2.25 |
| **823** | 3.72E-03 | 2.16 |
| **255** | 7.76E-03 | 2.1 |
| **569** | 4.16E-03 | 2.06 |
| **263** | 3.40E-03 | 2.03 |
| **573** | 3.92E-03 | 1.93 |
| **691** | 1.05E-04 | 1.88 |
| **910** | 2.19E-03 | 1.87 |
| **896** | 1.78E-03 | 1.86 |
| **616** | 3.69E-03 | 1.82 |
| **1391** | 3.07E-04 | 1.8 |
| **366** | 7.76E-03 | 1.8 |
| **562** | 9.04E-03 | 1.77 |
| **242** | 4.36E-05 | 1.72 |
| **826** | 4.30E-03 | 1.72 |
| **579** | 5.22E-03 | 1.72 |
| **970** | 9.22E-03 | 1.72 |
| **810** | 4.65E-03 | 1.68 |
| **886** | 7.99E-03 | 1.67 |
| **1222** | 1.96E-03 | 1.62 |
| **1086** | 8.00E-03 | 1.62 |
| **235** | 2.04E-04 | 1.61 |
| **269** | 4.22E-03 | 1.61 |
| **234** | 3.18E-03 | 1.55 |
| **937** | 9.95E-03 | 1.52 |
| **670** | 9.31E-03 | 1.51 |
| **901** | 8.50E-03 | 1.49 |
| **854** | 6.99E-03 | 1.48 |
| **1124** | 2.84E-03 | 1.4 |
| **494** | 5.80E-04 | 1.39 |
| **489** | 5.06E-03 | 1.35 |
| **534** | 5.89E-03 | 1.3 |
| **527** | 7.80E-03 | 1.26 |
| **897** | 9.27E-03 | 1.25 |
| **1125** | 6.07E-03 | -1.21 |
| **841** | 1.94E-03 | -1.24 |
| **386** | 9.76E-03 | -1.26 |
| **948** | 8.84E-04 | -1.32 |
| **943** | 1.71E-03 | -1.32 |
| **524** | 3.24E-04 | -1.4 |
| **1073** | 3.58E-04 | -1.42 |
| **912** | 6.23E-03 | -1.42 |
| **1084** | 5.77E-03 | -1.43 |
| **877** | 1.57E-03 | -1.44 |
| **1103** | 3.56E-03 | -1.44 |
| **1004** | 1.22E-03 | -1.45 |
| **972** | 7.19E-03 | -1.45 |
| **483** | 7.71E-03 | -1.46 |
| **1017** | 1.48E-03 | -1.47 |
| **334** | 2.39E-03 | -1.47 |
| **508** | 2.85E-03 | -1.47 |
| **390** | 4.46E-03 | -1.47 |
| **568** | 6.95E-04 | -1.5 |
| **326** | 2.10E-03 | -1.51 |
| **297** | 6.20E-03 | -1.53 |
| **329** | 1.22E-03 | -1.54 |
| **1043** | 4.75E-03 | -1.55 |
| **743** | 6.32E-03 | -1.6 |
| **1011** | 6.46E-03 | -1.65 |
| **591** | 3.85E-03 | -1.66 |
| **1010** | 3.05E-03 | -1.68 |
| **980** | 3.46E-03 | -1.71 |
| **1028** | 1.02E-04 | -1.73 |
| **1029** | 6.06E-03 | -1.73 |
| **944** | 9.80E-04 | -1.74 |
| **997** | 5.09E-05 | -1.75 |
| **1018** | 5.20E-04 | -1.76 |
| **477** | 6.88E-03 | -1.81 |
| **288** | 4.73E-03 | -1.82 |
| **996** | 8.17E-05 | -1.83 |
| **518** | 4.41E-03 | -1.83 |
| **1030** | 8.75E-03 | -1.83 |
| **1041** | 5.60E-03 | -1.84 |
| **1019** | 2.73E-04 | -1.87 |
| **1033** | 3.22E-04 | -1.92 |
| **1034** | 3.13E-03 | -1.93 |
| **993** | 5.57E-05 | -1.94 |
| **994** | 3.26E-03 | -2.06 |
| **1100** | 4.51E-04 | -2.09 |
| **721** | 2.15E-04 | -2.11 |
| **274** | 6.53E-03 | -2.37 |
| **1176** | 5.53E-03 | -2.47 |
| **942** | 1.10E-03 | -2.52 |
| **142** | 8.19E-04 | -2.56 |
| **795** | 7.93E-03 | -2.74 |
| **657** | 2.87E-05 | -8.29 |
|  |  |  |
